# Changes in Symptoms and General Well-being After Reversal of Roux-en-Y Gastric Bypass: A Questionnaire Survey

**DOI:** 10.1007/s11695-024-07321-2

**Published:** 2024-05-29

**Authors:** Sigrid B. Gribsholt, Lene R. Madsen, Inge M. Poulsen, Jens M. Bruun, Bjørn Richelsen

**Affiliations:** 1 Department of Endocrinology and Internal Medicine, Aarhus University Hospital, Palle Juul-Jensens Boulevard 165, 8200 Aarhus N, Denmark; 2 Steno Diabetes Center Aarhus, Aarhus University Hospital, Palle Juul-Jensens, Boulevard 99, 8200 Aarhus N, Denmark; 3 Department of Internal Medicine, Gødstrup Hospital, Hospitalsparken 15, 7400 Herning, Denmark; 4 Danish Diabetes Academy, Odense University Hospital, Kløvervænget 6, 5000 Odense C, Denmark; 5 Department of Surgery, Hvidovre Hospital, Kettegård Allé 30, 2650 Hvidovre, Denmark; 6 Department of Clinical Medicine, Aarhus University, Palle Juul-Jensens Boulevard 99, 8200 Aarhus N, Denmark

**Keywords:** Roux-en-Y gastric bypass, Reversal, Well-being, Symptoms

## Abstract

**Purpose:**

After Roux-en-Y gastric bypass (RYGB), few patients develop severe complications, which ultimately may require reversal of RYGB. We aimed to examine the effect of reversal of RYGB on symptoms and well-being.

**Materials and Methods:**

Via contact to medical and surgical departments treating patients with RYGB, we identified 18 patients, who had undergone reversal, 2009–2019. We conducted a Danish, nationwide questionnaire survey concerning symptoms before and after reversal of the RYGB including the patients’ own perceptions of their well-being.

**Results:**

Fourteen patients responded to the questionnaire (86% female; median age at RYGB, 36.2 years [IQR, 30.9–38.6 years]). The median time from RYGB to reversal was 5.8 years (IQR, 5.1–7.5 years). After RYGB, 13 patients (93%) reported abdominal pain, while 12 patients still had abdominal pain after reversal. Six out of 11 patients (45%) reported complete remission of dumping/post-bariatric hypoglycemia (PBH) after reversal. Malabsorption disappeared in 10 out of 11 patients (90%). Reversal had minor effect on neuropathy. The median weight loss from RYGB was 61 kg (IQR, 56–75 kg), while the median weight regain after reversal was 30 kg (IQR, 13–46 kg). Regarding the well-being, 72 of the patients felt better or much better after reversal.

**Conclusion:**

In total, 72% of the patients felt better or much better after reversal of RYGB, though some still had RYGB-related symptoms. The reversal relieved dumping/PBH and malabsorption, but not abdominal pain and neuropathy. Finally, half of the weight loss was regained after reversal. Reversal of RYGB may be an option in highly selected cases.

**Graphical Abstract:**

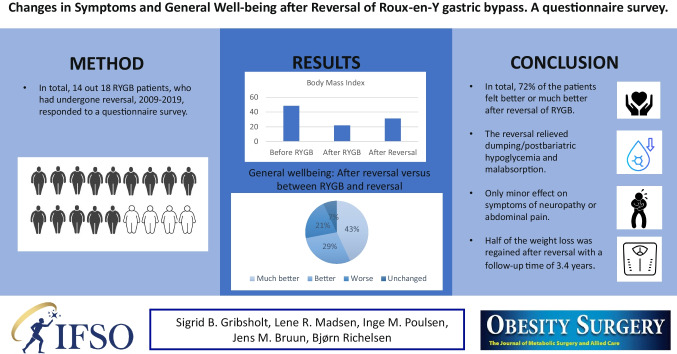

**Supplementary Information:**

The online version contains supplementary material available at 10.1007/s11695-024-07321-2.

## Introduction

Roux-en Y gastric bypass (RYGB) has well-documented positive effects in the treatment of severe obesity by inducing pronounced weight loss and reduction of obesity-related comorbidity such as type 2 diabetes mellitus [1, 2], cardiovascular risk factors [3], and mortality [4, 5]. The majority of patients treated with RYGB report increased quality of life, but some patients experience major side effects such as dumping/severe hypoglycemia, malnutrition, abdominal pain, and/or diarrhea [6]. These side effects are, for the majority, treatable with dietary changes and/or medical/surgical treatments. However, for a very small number of patients, reversal of the RYGB surgery may be the best or only available treatment possibility [7–9].

Previous studies on reversal of RYGB have mainly focused on surgical technique and safety associated with the surgical procedure itself [7, 8]. When RYGB is reversed, the mechanisms that initially led, e.g., to the weight loss and the surgery-derived positive effects may also be reversed, leading to weight regain. Moreover, the surgical reversal may by itself produce new symptoms or complications [8]. Our knowledge of the long-term outcomes of RYGB reversal in relation to symptom relief of the side effects, weight changes, and patient satisfaction following the procedure is very sparse. Such knowledge is important in order to establish the right indications for performing RYGB reversal and thereby improve our advice to patients suffering from severe symptoms/complications after RYGB.

To fill this gap, we aim to examine the consequences of reversal of RYGB with regard to weight changes, symptom relief, and overall satisfaction.

## Methods

### Participants

In Denmark, the Danish National Board of Health provides tax paid health care, including RYGB surgery and reversal [10]. RYGB reversal is a specialized treatment and is performed at three centers. From these centers, we identified 18 patients who had had reversal of their RYGB (2009–2019). We mailed the study questionnaire (Supplementary Fig. 1) to the patients. If no response, we reminded once by letter and thereafter by telephone. In total, three patients refused to complete the questionnaire, while one patient was unknown at the address given (Supplementary Fig. 1). Of the four patients who did not respond, two were female.

In total, approximately 16,000 patients have undergone RYGB in Denmark from 2008 to 2021 [11].

### Questionnaire

The questionnaire included items on height, weight, employment status, and different complications/symptoms before RYGB (pre-RYGB), after RYGB (post-RYGB), and after reversal of RYGB (post-reversal) (Supplementary Table 1, Supplementary Fig. 2). In order to grade the severity of symptoms, we asked patients if the symptom had led to any contact with the health care system. Thus, we constructed a symptom severity score system in patients with symptoms categorizing patients as follows: 1, no contact to the health system (1 point); 2, contact with general practitioner (2 points); 3, contact with outpatient specialized clinic (3 points); or 4, hospital inpatient admission (4 points). The participants were asked whether they have had the specific symptoms—(1) after RYGB but before reversal and (2) after reversal of RYGB. Symptoms of early dumping and reactive hypoglycemia could be difficult to separate; therefore, we pooled them into one group—the dumping/post-bariatric hypoglycemia (PBH) group. It should be emphasized that from our questionnaire, it is not possible to determine which symptoms lead to reversal surgery. Thus, a patient with severe PBH leading to reversal of RYGB may also have had some degree of abdominal pain etc. (Table 1).

**Table 1:** Characteristics of the 14 patients who had performed reversal of their RYGB. Prevalence and severity of symptoms before RYGB, after RYGB but before reversal of RYGB (post-RYGB), and after reversal of RYGB (post-reversal) from the questionnaire

	Pre-RYGB*	Post-RYGB**	Post reversal***
Weight, kg, median (IQR)	133 (120, 148)	61 (56, 75)	90 (79, 107)
Body mass index, kg/m^2^, median (IQR)	48.3 (43.3, 50.1)	21.9 (19.8, 26.0)	31.2 (26.9, 38.3)
Abdominal pain		13 (92.9%)	12 (85.7%)
Mean symptom severity score		3.8	3.3
Dumping		11 (78.6%)	6 (42.9%)
Mean symptom severity score		2.6	2.3
Neuropathy		7 (50.0%)	7 (50.0%)
Mean symptom severity score		3.4	2.9
Malabsorption		10 (71.4%)	3 (21.4%)
Mean symptom severity score		3.4	2.3
Your own opinion on your general wellbeing		**Post- vs pre-RYGB**	A**fter reversal vs. post-RYGB**
Much better			6 (42.9%)
Better			4 (28.6%)
Worse			3 (21.4%)
Unchanged			1 (7.1%)
Employment status			
Full time	7 (50%)	2 (14.3%)	1 (7.1%)
Part time	2 (14.3%)	1 (7.1%)	1 (7.1%)
Sick leave		5 (35.7%)	1 (7.1%)
Welfare payments			2 (14.3%)
Pension	4 (28.6%)	5 (35.7%)	8 (57.1%)
Other	1 (7.1%)	1 (7.1%)	1 (7.1%)

^*^Median age at time of RYGB surgery, 36.2 years (IQR, 30.9; 38.6 years)

^**^Median time from RYGB to reversal surgery, 5.8 years (IQR, 5.1; 7.5 years)

^***^Median follow-up time after reversal of RYGB, 3.4 years (IQR, 1.8; 4.4 years)

### Procedures/Investigations Before RYGB Reversal

The decision of performing surgical reversal of the RYGB was always made by a multidisciplinary team (surgeons, endocrinologists, dieticians, nurses etc.). Dependent of the specific complication/symptom, the patients were thoroughly investigated (e.g., CT of abdomen and laparoscopy), and all dietary and medical treatment options were exhausted (e.g., somatostatin analogues) in cases with severe PBH. All patients had a tube placed in the out-shunted stomach and were tube fed for 2–4 weeks in order to determine relief of symptoms by this procedure before submitted to surgical reversal.

### Surgical Technique for Reversal of RYGB

The gastrojejunostomy anastomosis was resected laparoscopically and reunion of the gastric pouch with the out-shunted gastric remnant was performed. The entero-entero anastomosis was divided with rejoining of the intestine in order to recreate the normal anatomy by anastomosing the Roux limb in continuity to the bilio-pancreatic limb without resection of intestine. The reversal technique was according to practice as described by Shoar et al. [8] where the Roux limb was reconnected in 57.2% of the patients and resected in 42.8% of the patients.

### Statistical Analyses

We tabulated characteristics of the patients at pre-RYGB, post-RYGB, and post-reversal. We also tabulated the prevalence of symptoms and calculated the mean symptom score for patients at pre-RYGB and post-RYGB; however, we performed no statistical testing for significance. Furthermore, we presented graphically the well-being pre-RYGB and post-RYGB.

According to Danish legislation, questionnaire studies do not require a separate permission from the Danish Scientific Ethical Committee [12]. The study was registered by the Danish Data Protection Agency (ref. no.: 1–16-02–80-19).

## Results

### Patient Characteristics and Body Weight

Of the 14 patients who responded to the questionnaire, 86% were female. The median age at time of RYGB was 36.2 years (IQR, 30.9; 38.6 years), while the median time from RYGB to reversal was 5.8 years (IQR, 5.1; 7.5 years) (Table 1). The median BMI was 48.3 kg/m^2^ (IQR, 43.3; 50.1 kg/m^2^) before RYGB and decreased to a nadir BMI of 21.9 kg/m^2^ (IQR, 19.8; 26.1 kg/m^2^) after RYGB. The median total weight loss from RYGB to nadir body weight before reversal was 61 kg (IQR, 56; 75 kg), corresponding to a median BMI of 31.2 kg/m^2^, while the median weight regain after reversal was 30 kg (IQR, 13; 46 kg) with a mean follow-up after the reversal of 3.4 years (IQR, 1.8; 4.4 years). Thus, about 50% of the weight loss obtained after RYGB was regained after reversal of the RYGB but the BMI was, however, still lower (31.2 kg/m^2^) than before RYGB surgery (48.3 kg/m^2^), corresponding to a post-reversal weight loss of 16.4 (IQR, 4.4; 22.4) BMI points.

One patient reported remission of pre-surgery type 2 diabetes mellitus, while two patients reported to be diagnosed with type 2 diabetes mellitus after reversal of RYGB (data not shown).

### Changes in RYGB-Related Symptoms

#### Abdominal Pain

As expected in this group, complications/symptoms were very frequent after RYGB (Table 1). Thus, 13 of the 14 patients (93%) reported to suffer from abdominal pain (both acute and chronic) after RYGB with a mean symptom score of 3.8. After reversal of RYGB, 12 patients still reported some degree of abdominal pain/abdominal discomfort, though the symptoms severity score had decreased to 3.3 (Table 1 and Supplementary Table 1).

#### Dumping/PBH

Eleven patients (79%) reported symptoms of dumping/PBH after RYGB (Table 1), with a mean severity symptom score of 2.6. After reversal of RYGB, five patients completely resolved, while six patients still reported dumping/PBH symptoms. However, the symptom severity score had decreased to 2.3 in these six patients (Table 1 and Supplementary Table 1).

#### Malabsorption and Other Symptoms

Of all patients, 10 patients (71%) reported symptoms of malabsorption (e.g., continuous weight loss/low weight, food intolerance) after RYGB with a mean symptom severity score of 3.4. Only three patients reported symptoms of malabsorption after reversal of the RYGB with a symptom severity score of 2.3.

Seven patients (50%) reported symptoms of neuropathy both after RYGB and after reversal. However, the symptom severity score decreased from 3.4 to 2.9 (Table 1).

### General Well-being and Employment Status

**Fig. 1:**
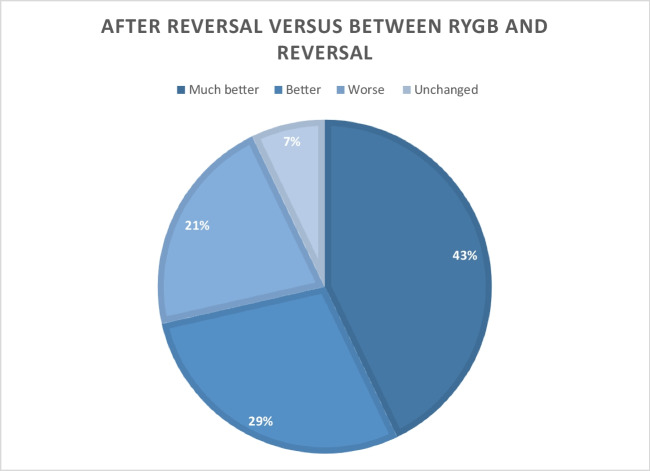
General well-being (*n* = 14)

Ten of the 14 patients (72%) reported to feel better or much better following RYGB reversal (Table 1 and Fig. 1). On the other hand, three patients reported that their symptoms deteriorated following reversal surgery. Regarding employment status, seven patients had a full-time job before RYGB, while two patients had a full-time job after RYGB. Only one patient had a full-time job after reversal (Table 1). Before RYGB, four of 14 patients (29%) received pension, which increased to eight (57%) after the RYGB reversal.

## Discussion

In this study, we focus on a small group of patients with severe symptoms after RYGB, leading to reversal of the procedure. Overall, 72% reported that they felt better or much better after reversal of RYGB, though some patients still had complications or symptoms post-reversal. Even though more than 70% felt better after the reversal surgery, this is not reflected in improved status of employment.

Symptoms of dumping and PBH disappeared in almost half of the patients, and the severity of dumping/PBH decreased in the rest. Symptoms of malabsorption almost completely disappeared, whereas reversal of RYGB had a minor effect on neuropathy-like symptoms: the number of patients with symptoms was unchanged, but the severity decreased. In the present study, the patients with abdominal pain before reversal had all been thoroughly examined and treated for anastomotic ulcers, internal herniation etc. Moreover, patients reporting abdominal pain also had co-existing symptoms such as PBH and malabsorption. Therefore, we cannot identify the main symptom and thereby reason for reversal surgery from our questionnaire. Nevertheless, among the patients with abdominal pain, 90% still reported some pain after reversal surgery. We can only speculate, if these symptoms stem from the original side effects or from side effects related to the reversal such as reflux, stenosis, or gastroparesis.

On the other hand, we observed a weight gain of 30 kg, corresponding to a weight regain of about 50% of the initially lost weight after reversal of the RYGB, which is in agreement with other studies [8, 9]. Notably, the post-reversal weight was still 16.4 BMI points lower than the weight before RYGB. Moreover, two patients developed type 2 diabetes mellitus after the reversal surgery most likely due to the weight gain. The RYGB-induced weight loss in this small group is, however, more pronounced than generally seen after RYGB [6]. Whether the more pronounced weight loss may be involved in the development of the post-RYGB complications, or on the other hand that patients with severe complications tended to lose more weight remains unsolved. Although the patients regained weight after the reversal of RYGB, the mean BMI was still lower after reversal than before RYGB after median 3.4 years of follow-up.

The reasons for reversal of the RYGB in our study were rather similar to previous studies [7–9] and included abdominal pain (of various reasons), dumping/PBH, and malabsorption. The reversal surgery had a pronounced effect on severe dumping/PBH, and although half of the patients still experienced symptoms after reversal, there were of a lesser extent. These findings are in agreement with recent studies reporting symptomatic improvement of hypoglycemia in most patients after reversal of RYGB [13]. Improvement of PBH is also expected based on the pathophysiological mechanisms of this symptom/complication, which is mainly related to the altered nutritional flow through the gastrointestinal tract after RYGB, which is normalized after the reversal [13, 14]. In Denmark, the consensus is that patients experiencing PBH are tube fed via the out-shunted stomach 2–4 weeks prior to the reversal. Remission of the hypoglycemic symptoms on tube feeding is a prerequisite for proceeding with reversal of their RYGB. Malabsorption (including food intolerance and low body weight) disappeared almost completely after reversal of the RYGB.

The number of patients reporting neuropathic symptoms was almost unchanged after reversal, indicating that reversal surgery may not be treatment of choice, and that neurological damages may be permanent.

RYGB is a commonly performed and generally safe procedure [15], but some well-known complications, such as food intolerance, malabsorption, dumping/PBH, and abdominal pain, may occur after RYGB. The side effects/complications are generally treatable with diet, medication, or surgery (e.g., internal herniation). However, these complications/symptoms can be challenging to manage and under such circumstances reversal of the RYGB can be considered [9]. We acknowledge that reversal of RYGB is a major operation reserved for the management of severe conditions or complications following RYGB. The procedure is complex, with the potential of weight gain and risk of relapse or deterioration of obesity-related comorbidities. Moreover, the effect of reversal surgery on rather diffuse symptom, “abdominal pain” is not straightforward, since most of these patients still have some complains after the reversal surgery. This suggests some restraint when considering reversal surgery to patients with abdominal pain as the only symptom.

The surgical technique can result in stenosis symptoms, which may be temporary because of healing and permanent if scar tissue is produced. The symptoms include reflux, vomiting, or pain after food intake with a feeling of blocking. Pyloric spasms leading to pain after food intake, a sense of bloating and vomiting, may occur because of vagal nerve damage either at the primary RYGB or at the reversal. Both complications may be diagnosed and treated by gastroscopy [8].

### Limitations

Our study is limited by the number of patients as only few patients have had reversal in Denmark. Another concern is that the questionnaire relied on self-reported symptoms elicited a long time after surgery. We did not ask about time relation between surgery and symptoms, since we considered that to be a risk of misclassification and that recall bias would be large.

Although our questions were comprehensive, focusing on the most common symptoms after RYGB surgery, we may, however, have missed some important symptoms. Moreover, the questionnaire was not validated in this cohort.

In total, 71% of patients completed the questionnaire. It is possible that the respondents had symptoms, which they were interested in addressing and therefore were more willing to answer a questionnaire. At the same time, respondents satisfied with the results of their operation may have wished to report their satisfaction.

Due to the low number of participants, we were not able to make a proper statistical analysis of the data which make it difficult to generalize the results.

## Conclusion

In total, 72% of the patients felt better or much better after reversal of RYGB, though some of them still have complains. The reversal was rather effective in improving symptoms of dumping/PHB and malabsorption, but the effect on abdominal pain was more doubtful. Reversal of RYGB had nearly no effects on neuropathy-like symptoms. Thus, reversal of RYGB may still be a useful treatment option in highly selected cases.

## Supplementary Information

Below is the link to the electronic supplementary material.

Supplementary file1 (DOCX 31 KB)
