# Supplementary material for: Changes in Symptoms and General Well-being After Reversal of Roux-en-Y Gastric Bypass: A Questionnaire Survey
Source: Obes Surg. 2024 May 29;35(1):33–9. doi: 10.1007/s11695-024-07321-2 (PMC11717792; doi:10.1007/s11695-024-07321-2)
Supplement: Supplementary file 1 — Supplementary file1 (DOCX 31 KB) [file 11695_2024_7321_MOESM1_ESM.docx]

**Supplementary Material**

**Supplementary Figure 1.** Flow chart of patients in the questionnaire survey

**Supplementary Figure 2.** Questionnaire sent to the participants

**Supplementary Table 1.** Numbers of patients (N=14) and severity score***** before and after reversal of RYGB

**Supplementary Figure 1.** Flow chart of patients in the questionnaire survey

18 patients who underwent RYGB reversal, 2016-2019

15 patients

3 refused to complete the questionnaire

1 with address unknown

14 patients

**Supplementary Figure 2.** Questionnaire sent to the participants

| Questionnaire  – for persons with reversed gastric bypass surgery | | | | | | | |
| --- | --- | --- | --- | --- | --- | --- | --- |
| Date of reply | | | \|  \|  \|  \|  \|  \|  \|  \|  \| \| --- \| --- \| --- \| --- \| --- \| --- \| --- \| --- \|   Ex.: 01012014 | | | | |
| Date of gastric bypass -surgery | | | \|  \|  \|  \|  \|  \|  \|  \|  \| \| --- \| --- \| --- \| --- \| --- \| --- \| --- \| --- \|   Ex.: 01012014 | | | | |
| Date of reversal of the gastric bypass | | | \|  \|  \|  \|  \|  \|  \|  \|  \| \| --- \| --- \| --- \| --- \| --- \| --- \| --- \| --- \|   Ex.: 01012014 | | | | |
| Your body weight and height | | | | | | | |
| What was your **max** weight before gastric bypass-surgery? | \|  \|  \|  \| \| --- \| --- \| --- \|   kg | | Which year? | | \|  \|  \|  \|  \| \| --- \| --- \| --- \| --- \|   Ex.: 2010 | | |
| What was your **min** weight after gastric bypass-surgery but before reversal? | \|  \|  \|  \| \| --- \| --- \| --- \|   kg | | Which year? | | \|  \|  \|  \|  \| \| --- \| --- \| --- \| --- \|   Ex.: 2010 | | |
| What is your current weight **after** reversal? | \|  \|  \|  \| \| --- \| --- \| --- \|   kg | |  | |  | | |
| What is your height?  *(without shoes)* | \|  \|  \|  \| \| --- \| --- \| --- \|   cm | |  | |  | | |
| **Questions regarding your employment and social situation.** | | | | | | | |
| **How was/is your employment situation?** (put a checkmark in each column) | | **Before** gastric bypass surgery | | **After** gastric bypass surgery but **before**  reversal | | **Current**  employment |  |
| Full-time employment | | □ | | □ | | □ |  |
| Part-time employment | | □ | | □ | | □ |  |
| Sick leave | | □ | | □ | | □ |  |
| Welfare payments | | □ | | □ | | □ |  |
| Retirement | | □ | | □ | | □ |  |
| Other | | □ | | □ | | □ |  |
| **Questions regarding smoking** | | | | | | | |
| **Have you ever smoked or do you smoke?** (put a checkmark in each column) | | **Before** gastric bypass surgery | | **After** gastric bypass surgery but **before**  reversal | | **Now –** after reversal |  |
| Daily | | □ | | □ | | □ |  |
| Yes, but not daily | | □ | | □ | | □ |  |
| No | | □ | | □ | | □ |  |

**The following questions are concerning the period before the gastric bypass surgery:**

| Have you had the following symptoms or diseases? | Did you have symptoms **before** gastric bypass? *(put a checkmark)* | If you had symptoms **before**  gastric bypass, did you have:  *(feel free to put more checkmarks)* |
| --- | --- | --- |
| Abdominal pain:  Abdominal pain and other symptoms from the stomach *(such as nausea, burping, or difficulty swallowing)* | Yes  □ | □No contact to the health care system  □Contact with general practitioner  □Contact with outpatient specialized clinic  □Hospital inpatient admission |
| Type 2 diabetes | Yes  □ | □No contact to the health care system  □Contact with general practitioner  □Contact with outpatient specialized clinic  □Hospital inpatient admission |
| Dumping and Hypoglycemia:  Dumping *(unpleasant symptoms from the stomach, nausea, cold sweats after a meal)* and/or hypoglycemia (trembling, shivering, discomfort half an hour to hours after a meal) | Yes  □ | □No contact to the health care system  □Contact with general practitioner  □Contact with outpatient specialized clinic  □Hospital inpatient admission |
| Neurological symptoms:  Symptoms from the nervous system  *(such as sensory disturbances, balance problems, or pain or reduced strength in arms and legs)* | Yes  □ | □No contact to the health care system  □Contact with general practitioner  □Contact with outpatient specialized clinic  □Hospital inpatient admission |
| Malabsorption:  Difficulty in absorbing food due to reduced intestinal function *(such as progressive weight loss)* | Yes  □ | □No contact to the health care system  □Contact with general practitioner  □Contact with outpatient specialized clinic  □Hospital inpatient admission |

**The following questions are concerning the period after the gastric bypass surgery and before the reversal:**

| Have you had the following symptoms or diseases? | Did you have symptoms **after** gastric bypass but **before** reversal? *(put a checkmark)* | If you had symptoms **after** gastric bypass but **before** reversal, did you have:  *(feel free to put more checkmarks)* |
| --- | --- | --- |
| Abdominal pain:  Abdominal pain and other symptoms from the stomach *(such as nausea, burping, or difficulty swallowing)* | Yes  □ | □No contact to the health care system  □Contact with general practitioner  □Contact with outpatient specialized clinic  □Hospital inpatient admission |
| Type 2 diabetes | Yes  □ | □No contact to the health care system  □Contact with general practitioner  □Contact with outpatient specialized clinic  □Hospital inpatient admission |
| Dumping and Hypoglycemia:  Dumping *(unpleasant symptoms from the stomach, nausea, cold sweats after a meal)* and/or hypoglycemia (trembling, shivering, discomfort half an hour to hours after a meal) | Yes  □ | □No contact to the health care system  □Contact with general practitioner  □Contact with outpatient specialized clinic  □Hospital inpatient admission |
| Neurological symptoms:  Symptoms from the nervous system  *(such as sensory disturbances, balance problems, or pain or reduced strength in arms and legs)* | Yes  □ | □No contact to the health care system  □Contact with general practitioner  □Contact with outpatient specialized clinic  □Hospital inpatient admission |
| Malabsorption:  Difficulty in absorbing food due to reduced intestinal function *(such as progressive weight loss)* | Yes  □ | □No contact to the health care system  □Contact with general practitioner  □Contact with outpatient specialized clinic  □Hospital inpatient admission |

**The following questions are concerning the period after the reversal:**

| Have you had the following symptoms or diseases? | Did you have symptoms **after** reversal? *(put a checkmark)* | | If you had symptoms **after** reversal, did you have:  *(feel free to put more checkmarks)* | |
| --- | --- | --- | --- | --- |
| Abdominal pain:  Abdominal pain and other symptoms from the stomach *(such as nausea, burping, or difficulty swallowing)* | Yes  □ | | □No contact to the health care system  □Contact with general practitioner  □Contact with outpatient specialized clinic  □Hospital inpatient admission | |
| Type 2 diabetes | Yes  □ | | □No contact to the health care system  □Contact with general practitioner  □Contact with outpatient specialized clinic  □Hospital inpatient admission | |
| Dumping and Hypoglycemia:  Dumping *(unpleasant symptoms from the stomach, nausea, cold sweats after a meal)* and/or hypoglycemia (trembling, shivering, discomfort half an hour to hours after a meal) | Yes  □ | | □No contact to the health care system  □Contact with general practitioner  □Contact with outpatient specialized clinic  □Hospital inpatient admission | |
| Neurological symptoms:  Symptoms from the nervous system  *(such as sensory disturbances, balance problems, or pain or reduced strength in arms and legs)* | Yes  □ | | □No contact to the health care system  □Contact with general practitioner  □Contact with outpatient specialized clinic  □Hospital inpatient admission | |
| Malabsorption:  Difficulty in absorbing food due to reduced intestinal function *(such as progressive weight loss)* | Yes  □ | | □No contact to the health care system  □Contact with general practitioner  □Contact with outpatient specialized clinic  □Hospital inpatient admission | |
| If you were to assess your overall well-being after reversal compared to the period with gastric bypass, how would you rate it...? | Much better  □ | Better  □ | Worse  □ | Unchanged  □ |

**Your own opinion:**

In your own words, what were the primary problems that led you to consider undergoing the reversal of the gastric bypass (in bullet points)?

________________________________________________________________________________________________________________________________________________________________________________________________________________________________________________________________________________________________________________________________________________________________________________________________________________________________________________________________________________________________________________________________________________________________________________________________________________________________________________________________________________________________________

In your own words: Briefly describe how you feel after the reversal surgical procedure, especially if you have experienced new problems/side effects that bother you?

____________________________________________________________________________________________________________________________________________________________________________________________________________________________________________________________________________________________________________________________________________________________________________________________________________________________________________________________________________________________________________________________________________________________________________________________________________________________________________________________________________________________________________________________________________________________________________________________________________________________________________________________________________________________________________________________________________________________________________________________________________________

**Supplementary Table 1.** Numbers of patients (N=14) and severity score***** before and after reversal of RYGB

| **Symptoms** | **Before reversal of RYGB , No.** | **After reversal of RYGB. No.** |
| --- | --- | --- |
| **Abdominal pain** |  |  |
| No | 1 | 2 |
| Yes, all | 13 | 12 |
| Yes, no health care contact |  |  |
| Yes, contact to general practitioner |  | 1 |
| Yes, outpatient contact | 2 | 6 |
| Yes, inpatient contact | 11 | 5 |
| Score/no. of patients with symptoms | 50/13 = 3.8 | 40/12 = 3.3 |
| **Dumping/PBH** |  |  |
| No | 3 | 8 |
| Yes, all | 11 | 6 |
| Yes, no health care contact | 3 | 1 |
| Yes, contact to general practitioner | 1 | 1 |
| Yes, outpatient contact | 4 | 2 |
| Yes, inpatient contact | 3 | 2 |
| Score/no. of patients with symptoms | 29/11= 2.6 | 17/6 =2.3 |
| **Neuropathy** |  |  |
| No | 7 | 7 |
| Yes, all | 7 | 7 |
| Yes, no health care contact |  | 1 |
| Yes, contact to general practitioner | 1 |  |
| Yes, outpatient contact | 2 | 5 |
| Yes, inpatient contact | 4 | 1 |
| Score/no. of patients with symptoms | 24/7 = 3.4 | 20/7 = 2.9 |
| **Malabsorption** |  |  |
| No | 4 | 11 |
| Yes, all | 10 | 3 |
| Yes, no health care contact | 1 | 1 |
| Yes, contact to general practitioner | 1 | 1 |
| Yes, outpatient contact | 1 |  |
| Yes, inpatient contact | 7 | 1 |
| Score/no. of patients with symptoms | 34/10 = 3.4 | 7/3 = 2.3 |

PBH; post bariatric hypoglycemia

*Severity of symptoms; No = no symptoms, Yes, no contact to the health system =1 point; yes, contact with general practitioner = 2 points; yes, contact with outpatient specialized clinic = 3 points; yes, hospital inpatient admission =4 points.
